# High systemic gentamicin levels and ototoxicity after implantation of gentamicin beads in a 70-year-old man—a case report

**DOI:** 10.3109/17453670903487032

**Published:** 2009-12-04

**Authors:** Paul A G de Klaver, Jan de Koning, Rob P A Janssen, Luc J J Derijks

**Affiliations:** ^1^Departments of Clinical Pharmacy; ^2^Departments of Intensive Care; ^3^Departments of Orthopedic Surgery and Traumatology, Máxima Medical Center, Veldhoven, the Netherlands

## Introduction

Gentamicin beads are used for musculoskeletal infections. Gentamicin is released gradually from the beads, and high gentamicin concentrations are achieved only locally. Side effects of gentamicin are rarely to be expected with gentamicin beads since only low amounts of gentamicin are released from the beads, resulting in extremely low serum concentrations of the antibiotic (Septopal SPC, Wahlig et al. 1978, Walenkamp et al. 1986). Only one case of prolonged raised gentamicin serum levels from gentamicin beads has been described to date, but no adverse effects were reported in that case (Walenkamp and Vree 1981). We present another case with ototoxicity also.

### Case

A 70-year-old man had a total knee replacement performed because of osteoarthritis. He had a history of atrial fibrillation, heart failure, mitral regurgitation, chronic obstructive pulmonary disease, diabetes mellitus, and he had no known drug allergies. He had never complained of hearing loss.

The postoperative course was complicated by paralytic ileus, perforation of the small bowel, and acute renal failure (which resolved with continous veno-venous hemofiltration) for which intensive care was necessary from day 7 to day 18 postoperatively. The patient was re-admitted to the intensive care unit from day 21 to 23, for bleeding complications. Hearing loss, which reversed after a few days, was reported on day 23, 11 days after a 400-mg single preoperative intravenous dose of gentamicin. The patient was admitted to the intensive care unit again—from day 64 to day 67—for renal failure caused by fluid depletion, which resolved after filling. On day 112, the patient was admitted to the intensive care unit for respiratory insufficiency after surgery for partial small bowel resection. During this stay in intensive care, surgery for fascia dehiscence after laparotomy and surgery for multiple enterocutaneous fistulas were performed. A *Staphylococcus aureus* sepsis on day 157, caused by a purulent arthritis of the left hip that was diagnosed on day 161, was treated by arthrotomy drainage and irrigation with implantation of 120 gentamicin beads on day 162. A Girdlestone arthroplasty was performed on day 176 and the gentamicin beads were changed. At the time of implantation of the gentamicin beads and thereafter, the patient had a normal renal function (estimated MDRD of 65 mL/min/1.73 m^2^, reference value > 60 mL/min/1.73 m^2^).

Medications during this 94-day period on intensive care included furosemide (20 mg i.v. twice daily initially, and later increased to 500 mg every 24 hours), ceftazidim (1,000 mg i.v. 3 times daily), flucloxacillin (continuously, 12 g i.v. daily), ciprofloxacin (400 mg i.v. twice daily), potassium chloride (i.v. and per os), metoclopramide (10 mg i.v. 3 times daily), buprenorphine (70 mcg/h transdermal patch every 3 days), midazolam (i.v.), dalteparin (7,500 IE subcutaneously twice daily), fentanyl (i.v.), olanzapine (5 mg per os, initially twice daily but later reduced to once daily), acenocoumarol (per os), norepinephrine (i.v.), gabapentin (300 mg per os, initially once daily but later increased to 3 times daily) and spironolactone (25 mg per os once daily).

10 days after implantation of the gentamicin beads (day 172), the patient complained about sudden severe hearing loss and the gentamicin level was 0.7 mg/L. The reference value for trough gentamicin levels after gentamicin infusion is < 0.5 mg/L. The levels that followed were: 0.9 mg/L (day 179), 0.9 mg/L (day 181), 1.0 mg/L (day 187), 0.7 mg/L (day 194), and 0.4 mg/L (day 201). Gentamicin concentrations were detectable for 4 weeks, and were above the reference value for 3 weeks (Figure). During this period, the patient had not been treated with gentamicin through any other route. He died in the intensive care unit on day 216. No obduction was performed.

**Figure:**
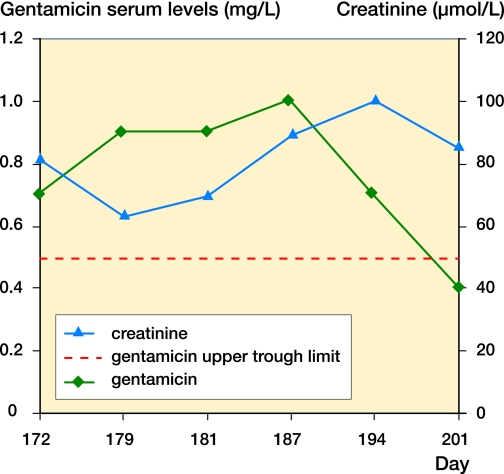
Systemic gentamicin concentrations and creatinine concentrations. Total knee replacement was performed on day 1. On day 162, 120 gentamicin beads were implanted. They were changed on day 176.

## Discussion

### Pharmacokinetics

Gentamicin-polymethylmethacrylate (gentamicin-PMMA) beads are balls of bone cement that release gentamicin locally at initially high concentrations, followed by a period of constant release for up to about 80 days. Systemically, only extremely low concentrations are detectable: generally, serum gentamicin levels remain below 0.1 mg/L. In daily clinical practice, serum levels of 0.4 mg/L and lower can be regarded as immeasurable. The blood-bone barrier, which gives difficulty in achieving therapeutic concentrations locally with systemic administration, prevents high serum concentrations with local therapy (Walenkamp et al. 1986). Renal clearance of gentamicin mainly depends on glomerular filtration, with only a small amount of tubular reabsorption (Chiu et al. 1976).

Studies have been published on the pharmacokinetics of gentamicin beads, but with a relatively small number of patients.

Gentamicin levels measured in 5 patients with 48–360 beads were measurable only in 1 patient (with 360 beads implanted). After a peak level of 1.8 mg/L on day 1, a plateau level of 0.4 mg/L was reached. This patient had a plasma creatinine level of 73 µmol/L (reference value 60–110 µmol/L). The release and excretion depended on vascularization of the surrounding tissues: excretion was faster when beads were placed in muscle tissue than when they were placed in sclerotic bone tissue (Walenkamp et al. 1986). The largest study involved 41 patients. Only traces of gentamicin were found, even in patients with the largest numbers (80–180) of beads (Wahlig et al. 1978). The only case report of raised gentamicin serum levels with gentamicin beads decribes the implantation of 150 beads in a patient with creatinine clearance of 1 mL/min (reference value > 60 mL/min). The gentamicin level in this patient was 3 mg/L and decreased to 2 mg/L. The duration of raised gentamicin levels was not described in this case report (Walenkamp and Vree 1981). No adverse effects have been reported with gentamicin beads.

In our case, the serum gentamicin levels were measurable for more then 3 weeks. This is unique; there have been no reports of gentamicin levels of this magnitude for such a long time and caused by gentamicin beads alone. Moreover, in our case there was no indication of renal failure during the period of raised gentamicin levels, and the amount of gentamicin beads implanted was lower than in the cases of raised gentamicin serum levels described above. The cause of the altered gentamicin pharmacokinetics may have been an increased uptake through increased local blood flow at the site of the wound, or possibly an impaired blood-bone barrier.

### Ototoxicity

All aminoglycosides can damage cochlear and vestibular organs. Cochleotoxicity presents as bilateral sensorineural hearing loss, initially affecting the high frequencies. Early toxicity often goes unnoticed, since the frequencies affected initially fall outside the range of speech (Rizzi and Hirose 2007, Seligmann et al. 1996). Damage then progresses to lower frequencies, including frequencies in the human speech range (Forge and Schacht 2000). Since gentamicin clearance from the inner ear is slow, ototoxicity may sometimes become manifest after some days or weeks (Bates et al. 2002).

Aminoglycoside-induced hearing loss is permanent in approximately half of all patients affected. It may occur by any administration route, but has not been described with gentamicin beads (Seligmann et al. 1996). In 2 audiometric studies in patients with implanted gentamicin beads, no ototoxicity was found (Haydon et al. 1993, Walenkamp et al. 1987). Even though there is no clear association between ototoxicity and aminoglycoside levels, raised levels could increase the risk of ototoxicity (Rizzi and Hirose 2007).

In our case, sudden bilateral hearing loss occurred ten days after implantation of the beads, and had progressed to frequencies in the human speech range. The patient had previously experienced reversible hearing loss 12 days after a 400-mg intravenous dose of gentamicin, and he may have been extremely sensitive to the ototoxic properties of gentamicin.

Use of the Naranjo adverse drug reaction probability scale (Naranjo et al. 1981) indicates a probable relationship between hearing loss and gentamicin beads: this adverse reaction has previously been reported with gentamicin in the literature. The timeline of implantation was consistent with the appearance of the adverse effect. A rechallenge has led to hearing loss and toxic levels of gentamicin were found in serum.

Risk factors that might possibly have been involved in our case were poor nutritional state, age, bacteremia, duration of measurable gentamicin serum levels of more then 10 days, or a genetic predisposition (Seligmann et al. 1996, Forge and Schacht 2000, Bates et al. 2002). Genetic predisposition was not evaluated in our patient. The combination with the loop diuretic furosemide may also have contributed, since this combination may lead to total deafness—even under conditions in which neither drug alone causes a hearing deficit (Forge and Schacht 2000). The furosemide dose used to stimulate diuresis in our case was fairly high, but not unusually high.

## References

[CIT0001] Bates D, Beaumont S, Baylis B (2002). Ototoxicity induced by gentamicin and furosemide. Ann Pharmacother.

[CIT0002] Chiu P, Brown A, Miller G, Long J (1976). Renal extraction of gentamicin in anesthetized dogs. Antimicrob. Agents Chemother.

[CIT0003] Forge A, Schacht J (2000). Aminoglycoside antibiotics. Audiol Neurootol.

[CIT0004] Haydon R, Blaha J, Mancinelli C, Koike K (1993). Audiometric thresholds in osteomyelitis patients treated with gentamicin-impregnated methylmethacrylate beads (Septopal). Clin Orthop.

[CIT0005] Naranjo C, Busto U, Sellers E, Sandor P, Ruiz I, Roberts E, Janecek E, Domecq C, Greenblatt D (1981). A method for estimating the probability of adverse drug reactions. Clin Pharmacol Ther.

[CIT0006] Rizzi M, Hirose K (2007). Aminoglycoside ototoxicity. Curr Opin Otolaryngol Head Neck Surg.

[CIT0007] Seligmann H, Podoshin L, Ben-David J, Fradis M, Goldsher M (1996). Drug-induced tinnitus and other hearing disorders. Drug Saf.

[CIT0008] Septopal SPC http://db.cbg-meb.nl/IB-teksten/h07618-h12500.pdf.

[CIT0009] Walenkamp G, Vree T (1981). Treatment of a patiënt with impaired renal function with gentamicin-PMMA beads. Arch Orthop Trauma Surg.

[CIT0010] Walenkamp G, Vree T, Rens T (1986). Gentamicin-PMMA beads. Clin Orthop.

[CIT0011] Walenkamp G, Vree T, Guelen P, Jongman-Nix B, Huygen P (1987). Side effects of gentamycin following implantation of PMMA containing gentamycin in the form of bead chains. Aktuelle Probl Chir Orthop.

[CIT0012] Wahlig H, Dingeldein E, Bergmann R, Reuss K (1978). The release of gentamicin from polymethylmethylacrylate beads. J Bone Joint Surg (Br).

